# Fractional CO_2_ laser treatment of post-surgical nasal scars: a retrospective analysis on 8 patients

**DOI:** 10.1007/s10103-026-04827-2

**Published:** 2026-05-02

**Authors:** Emanuele Maria Cipollini, Luca Mariano, Cristiano Morini, Alessandro Clementi, Luca Guarino, Marco Gratteri, Elena Zappia, Annunziata Dattola, Steven Paul Nisticò, Giovanni Cannarozzo

**Affiliations:** 1 University of Florence, Florence, Italy; 2 University of Modena and Reggio Emilia, Modena, Italy; 3 Sapienza University of Rome, Rome, Italy

**Keywords:** Fractional CO₂ laser, Nasal scar, Basal cell carcinoma

## Abstract

Surgical scars of the nasal region following basal cell carcinoma (BCC) excision often present aesthetic and functional challenges, particularly the “trap-door” deformity related to flap reconstruction. Fractional CO₂ laser represents a promising therapeutic option to improve scar quality through collagen remodeling and dermal reorganization. This retrospective study analysed eight patients (six males and two females, mean age 71 ± 10 years) with nasal post-surgical scars after BCC excision and flap reconstruction. All patients underwent two sessions of fractional CO₂ laser in Deep Pulse or High Pulse modes, with a two-month interval between treatments. Clinical outcomes were evaluated using the Patient and Observer Scar Assessment Scale (POSAS) and three-dimensional profilometry with the Antera 3D system before treatment and two months after the last session. A statistically significant improvement was observed in both patient-reported (overall opinion *p* = 0.0007; thickness *p* = 0.03) and observer-reported parameters (vascularity *p* = 0.007; thickness *p* = 0.005; pliability *p* = 0.02; overall opinion *p* = 0.00004). Antera 3D analysis showed a mean reduction in scar depression of 23.5% (*p* = 0.01). All patients developed transient erythema and edema that were resolved within a week. Fractional CO₂ laser proved to be a safe and effective method to improve nasal post-surgical scars, enhancing texture, vascularity, and contour.

## Introduction

Basal cell carcinoma (BCC) is the most frequent cutaneous neoplasm, constituting more than 80% of non-melanocytic skin cancers. It originates from the basal cells of the epidermis and is characterised by slow growth and limited metastatic capacity, although it can result in extensive local tissue destruction if not adequately treated [1]. The aesthetic and functional consequences are particularly relevant when BCC affects the facial district, the most common site of onset, with a marked predilection for the nasal region, an area of high cumulative exposure to ultraviolet radiation [2, 3]. BCC of the face, and particularly that located nasally, is classified as a site at medium to high risk of recurrence. Consequently, treatment of BCC of the nose, which is considered a high-risk location, requires a radical surgical approach that ensures complete eradication of the neoplasm while preserving nasal function and aesthetics [4]. The nose represents a central component of the aesthetic and functional balance of the face, organized into distinct aesthetic subunits whose integrity is critical for facial harmony; even minor morphological alterations can significantly affect the overall perception of the face [5, 6].

The nose plays a central role both functionally and psychologically. Its morphology influences body image and self-esteem. Alterations perceived as defects can reduce emotional well-being. Surgical treatment is often associated with an improvement in quality of life [7]. In nasal basal cell carcinoma (BCC), complete excision must therefore be combined with respect for functional and aesthetic units. Mohs micrographic surgery (MMS) allows for precise tumour removal. It reduces the risk of recurrence. It preserves healthy tissue. It facilitates the reconstruction of the surgical defect. For these reasons, it is considered the gold standard in the treatment of BCC [8, 9]. The nasal skin has unique characteristics. Skin thickness varies between the bridge, tip and wings. The distribution of sebaceous glands is uneven. These features change with age and influence healing and scar response [10–12].

The ‘trap-door effect’ is a common complication of flap scars. It is favoured by the curved surface of the nose. It is caused by tissue tension, oedema and scar contraction [13].

Nasal defects can be reconstructed using various surgical techniques, sometimes in combination. Early scar management is crucial to optimise the aesthetic outcome, and therapeutic strategies include laser treatment. Several laser systems have been employed in the treatment of nasal surgical scars after excision of basal cell carcinoma, including ablative devices, such as CO₂ and Er: YAG, and non-ablative devices, such as fractional lasers or pulsed dye lasers. These technologies aim to improve scar texture, reduce erythema and dyschromia, and promote collagen remodelling, highlighting the need for a personalised treatment approach based on the scar’s characteristics.

Surgical incisions inevitably lead to scar formation. However, the clinical objective is to obtain, at the end of maturation, a minimally visible scar outcome that is flat and chromatically uniform with the surrounding skin. The final outcome depends on multiple factors, including the planning of the incision, management of tension during closure and appropriate postoperative care [14–20]. In this context, the management of post-surgical nasal scarring remains a major clinical challenge, especially in terms of aesthetic outcome.

The present retrospective study evaluates the efficacy of fractionated CO₂ laser in the treatment of nasal surgical scars. The analysis was conducted using the Antera 3D system, which allows a quantitative and objective assessment of scar changes over time [21].

## Materials and methods

A total of 8 patients (6 males and 2 females, mean age 71.1 ± 10.6) were included in the analysis (demographic characteristics in Table 1).

We included patients who had undergone laser procedures and had completed the required documentation; informed consent was obtained for the acquisition of photographic images and for fractional CO2 laser treatment.

On average, they had undergone surgical excision in the nasal area within the past 18 months at Piero Palagi Hospital (Florence, Italy), with histopathological confirmation of basal cell carcinoma. surgical removal for basal cell carcinoma (BCC). Furthermore, the surgical site had to be reconstructed using a skin flap technique, and the resulting scar or flap needed to display signs of atrophy (5 scars) or the trap-door phenomenon (3 scars). Patients whose defects were closed by direct suturing or skin grafts were excluded, as these approaches are less likely to result in the trap-door effect.

**Table 1:** Demographic characteristics, flap site, and flap type of the 8 treated patients

No.	Sex	Age (years)	Involved Nasal Subunit	Flap Type	Time Since Surgery (months)
1	M	84	Ala–lateral sidewall	Advancement flap	9
2	M	49	Ala–lateral sidewall	Spiral flap (transposition flap)	12
3	M	76	Dorsum–lateral sidewall	Bilobed flap (transposition flap)	8
4	M	77	Lateral sidewall–tip	Rieger flap (Combined transposition and advancement flap)	7
5	M	82	Dorsum–lateral sidewall	Bilobed flap (transposition flap)	14
6	F	66	Ala–lateral sidewall	Nasal–cheek junction (transposition flap)	10
7	F	70	Lateral sidewall–ala	Horn flap (Myocutaneous island flap)	13
8	M	65	Lateral sidewall–dorsum	Bilobed flap (transposition flap)	9

The treated area was cleansed with saline solution, dried, and subsequently treated with fractional CO₂ laser (SmartXide² laser, DEKA-M.E.L.A., Calenzano, Florence), using either HP (High Pulse) or DP (Deep Pulse) mode as needed, with a single pass and approximately 20% overlap. The size and shape of the laser spot were adjusted according to the size and shape of the scar.

**Fig. 1:**
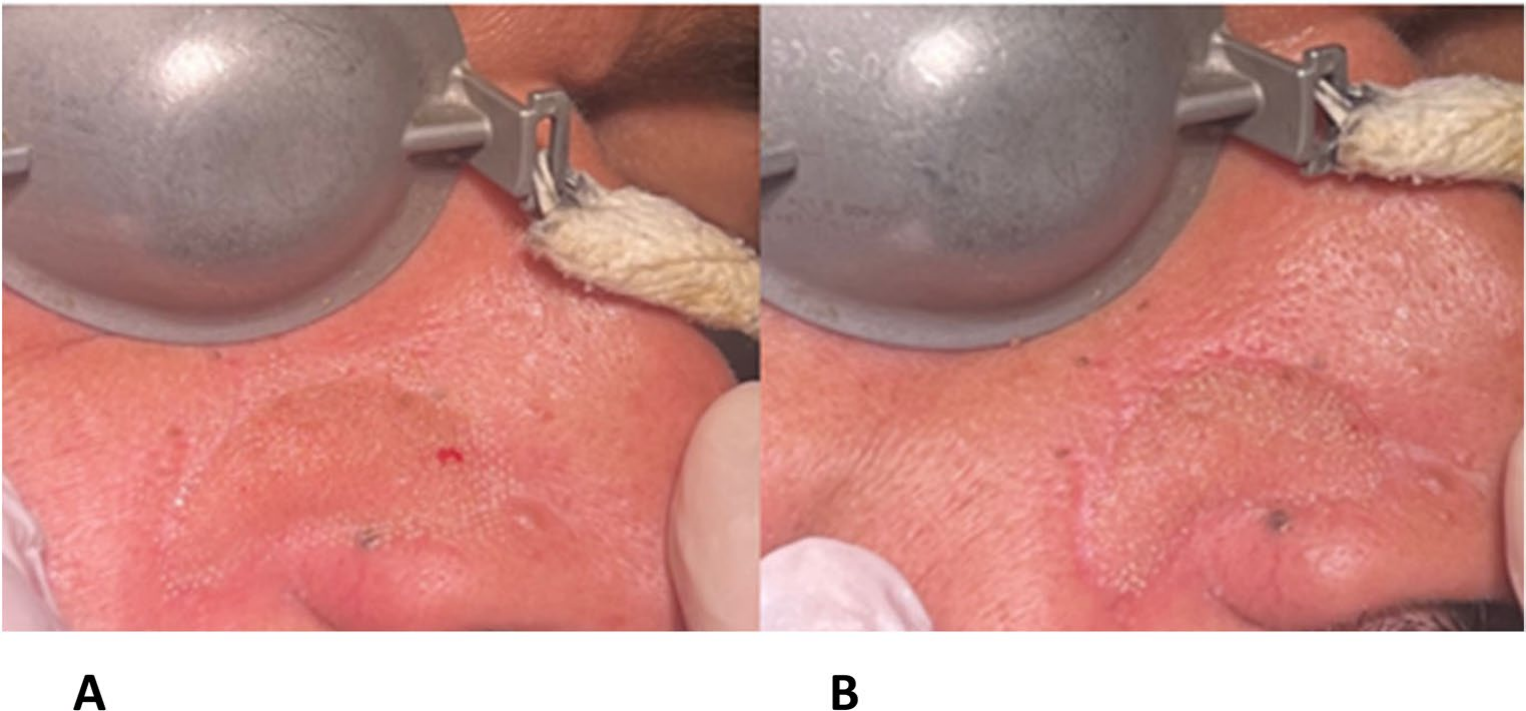
Laser treatment of a patient. In A, application of fractional CO₂ laser in *Deep Pulse* mode at the level of the surgical suture. In B, application of fractional CO₂ laser in *High Pulse* mode at the level of the flap elevated above the surrounding tissue planes

All patients were treated using the following parameters:

- **Depressed scars: Deep pulse mode (DP), power 15–18 W**, scan time 1800 µs, spacing 500 μm; density 13.3%, fluence 5.5–7 J/cm², pulse energy 40–60 mJ (Fig. 1A).
- **Any surrounding elevated skin areas**: High pulse mode (HP), power 15–18 W, spacing 500 μm; density 13.3%, fluence 5.5–7 J/cm², pulse energy 40–60 mJ (Fig. 1B).

Immediately afterward, cold-moist compresses were applied and changed every 2 min, followed by the application of a Comfeel dressing, which the patient replaced every 48 h for a period of two weeks. Sun exposure was highly avoided for the first 30 days after laser treatment.

Each patient received two laser treatments, with a two-month delay between them. The last examination was done two months after the treatment program had been completed.

 The POSAS Score (Patient and Observer Scar Assessment Scale) was utilized as a clinical instrument to assess scars based on both their physical qualities and the patient’s subjective feeling [22]; each parameter is rated on a scale from 1 to 10, where 1 represents normal skin and 10 indicates the most severe possible scarring issue. This assessment was carried out both at the start of the treatment (T0) and two months after it concluded (T2).

Photographic acquisitions were performed using a digital camera with polarized flash and were saved in a digital archive before and after each laser session, as well as two months following the last one. The same camera was utilized with the same technical parameters, dual flash, and ambient lighting conditions under control to guarantee image standardization. Additionally, a remote image overlay system was implemented to ensure a consistent distance between shots taken over time.

 In order to guarantee the most impartial assessment of scar improvement, a digital evaluation of the scar (depth, volume, and density) was carried out using an optical analysis device that employs multispectral light sources with three-dimensional mapping of the skin surface (wrinkles, pigmentation, vascularization, and texture), along with an analysis of the depression present at the level of the scar (Antera 3D, MIRAVEX, Dublino). With this technology, it is possible to accurately monitor any improvement or worsening of skin depression following fractional CO₂ laser treatment, allowing for a percentage-based evaluation of therapeutic efficacy over time [21].

The Antera evaluation was carried out before the first treatment session (T0), before the second session (T1), and two months after the end of the treatment (T2).

Due to the convex shape of the nose, the scar often involves different planes, making it difficult to assess the entire scarred area in a single frame. Therefore, in such cases, the assessment focused on the portion of the scar with the most severe features to evaluate treatment effectiveness. A color scale is used to indicate the degree of depression, ranging from yellow (mild depression) to purple (severe depression). Using dedicated software, it is possible to manually select the area to be analyzed in the T0 frame, excluding any physiological skin depressions and including only those within the scarred region. The software then automatically captures the same area in the T2 frame, ensuring an accurate comparison of the exact treatment zone for the report.

Utilizing the paired t-test, statistical analysis was conducted on the average differences between pre- and post-treatment values for the different POSAS Score parameters and for Antera images.

 A statistically significant P-value was defined as less than 0.05.

## Results

**Fig. 2:**
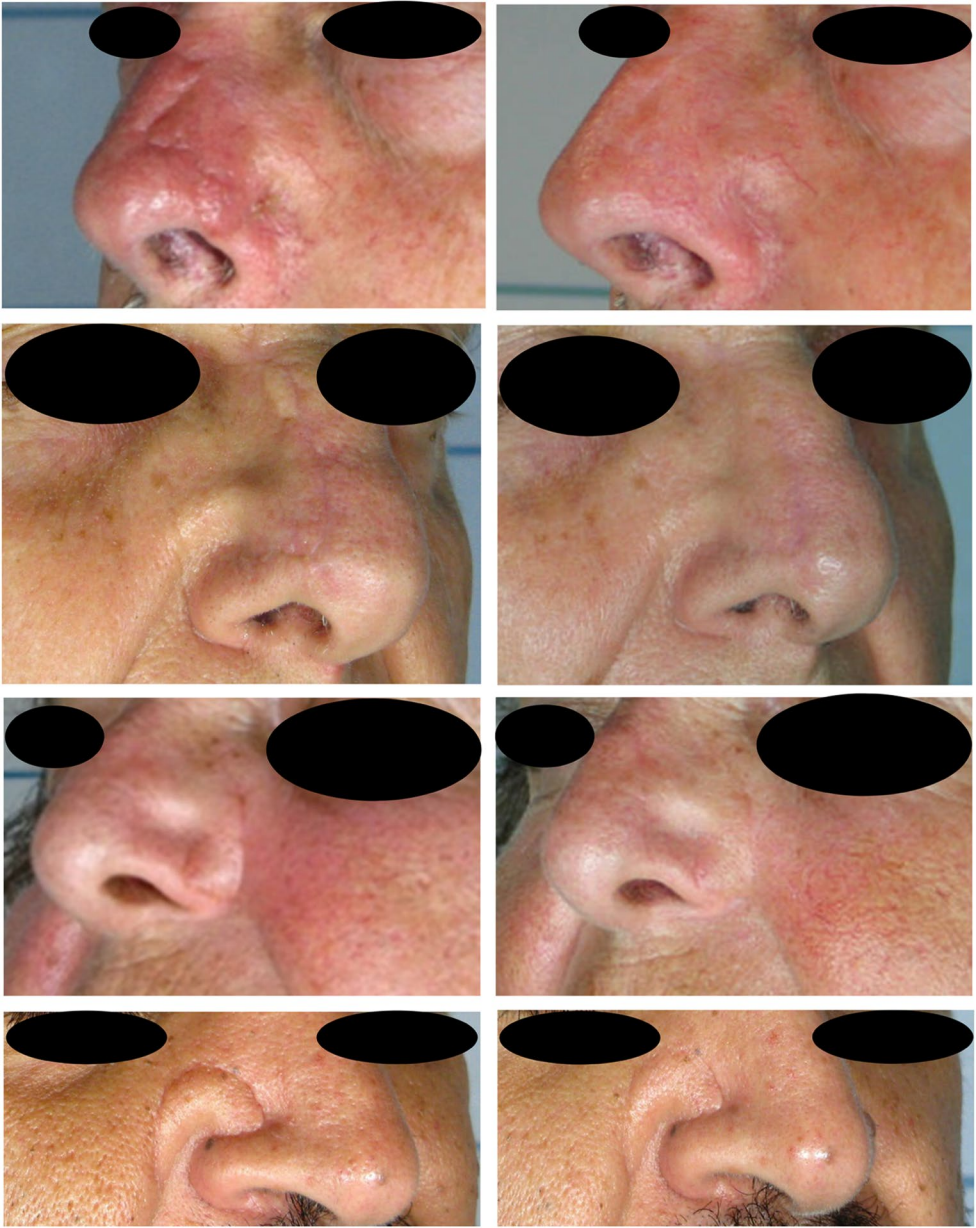
Clinical photographic evaluation of patient at the beginning of the first session (T0) (panels on the left) and two months after the end of the protocol (T2) (panels on the right)

**Fig. 3:**
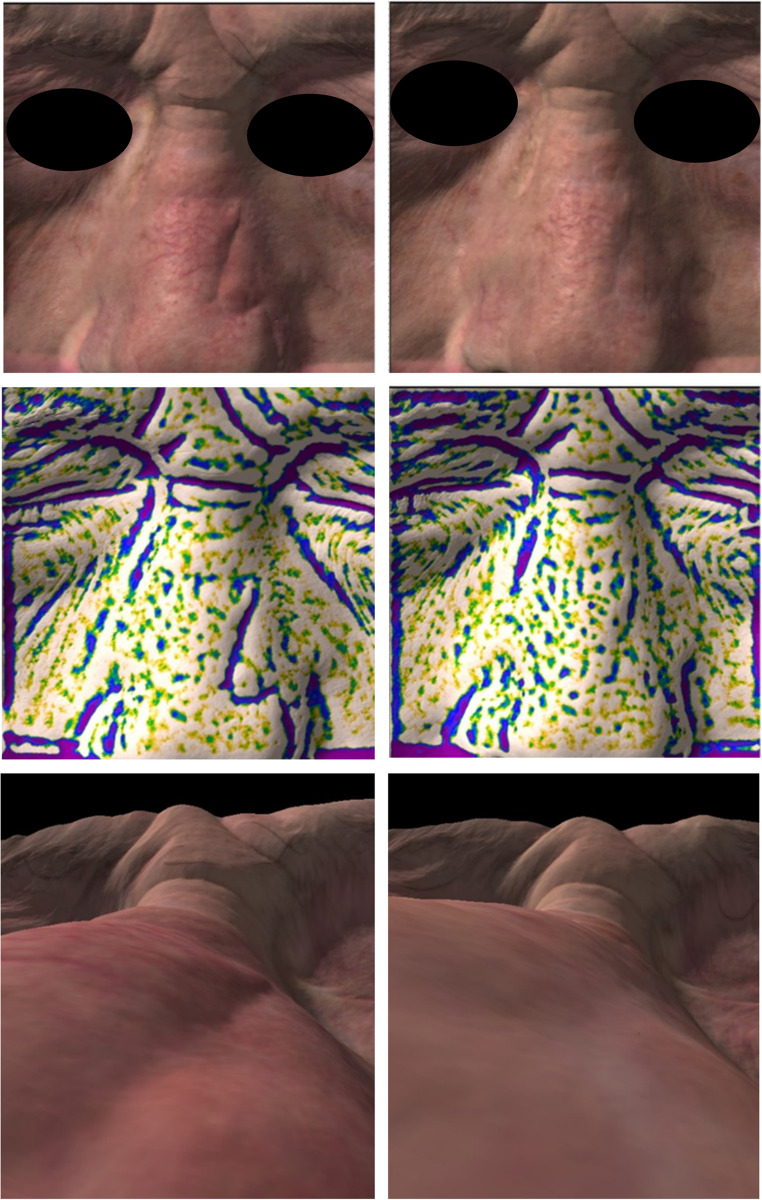
Frames captured using Antera 3D. Top image panel: evaluation of three-dimensional profilometry at T0 (left panel) and at T2 (right panel). Central image panel: evaluation of small depressions (0.1–1 mm) at T0 (left panel) and at T2 (right panel).Bottom image panel: sagittal section frame using Antera 3D of the surgical scar at T0 (left panel) and at T2 (right panel)

All patients underwent two sessions of fractional CO₂ laser treatment, spaced two months apart and the results indicated a general improvement in the scar’s appearance (Figs. 2 and 3) and functionality.

**Fig. 4:**
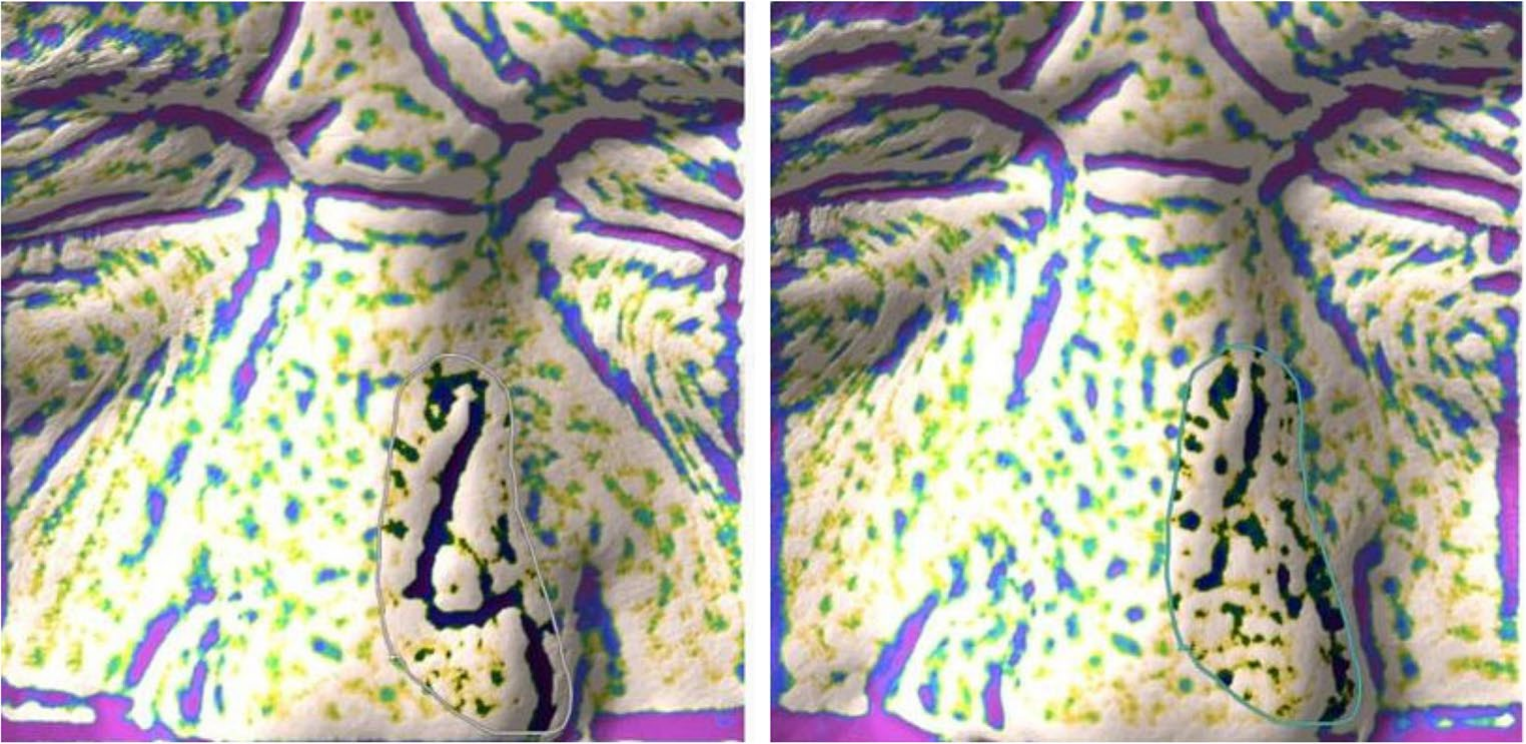
Manual selection of the depressed area to be assessed using Antera 3D

It is feasible to manually choose the region of the T0 picture to be examined, removing any physiological skin depressions and only include those associated with the scar area, thanks to specialized software (Fig. 4).

The perception of scar thickness (p-value 0.02) and the overall evaluation of the scar (p-value 0.0007) showed a statistically significant improvement among the different POSAS Patient scale criteria. The two patients who reported itching before treatment had an improvement in their symptoms at the conclusion of the regimen, however this improvement was not statistically significant (Table 2).

Vascularity (p-value 0.007), thickness (p-value 0.005), elasticity (p-value 0.02), and the overall evaluation of the scar (p-value 0.00004) were the POSAS Observer scale parameters that showed the most statistically significant improvement (Table 3).

The three-dimensional Antera evaluation showed a significant reduction in scar depression. Specifically, the results of the paired t-test indicate a statistically significant and meaningful difference between T0 (M = 6.65 mm³; SD = 2.39) and T2 (M = 5.04 mm³; SD = 1.93), t(7) = 4, *p* = 0.01 (Table 4). The mean volumetric difference of the scar pre/post treatment was−1.61 mm³.

**Table 2:** POSAS patient parameters for the eight treated patients at T0 and T2

No.	Pain	Itching	Color	Stiffness	Thickness	Irregularity	Overall opinion
1	1/1	1/1	3/2	1/1	3/2	1/1	3/2
2	1/1	1/1	1/1	4/2	4/3	1/1	3/2
3	1/1	4/2	1/1	1/1	1/1	2/1	2/1
4	1/1	1/1	1/1	1/1	1/1	1/1	1/1
5	1/1	1/1	1/1	1/1	1/1	1/1	1/1
6	1/1	1/1	2/1	2/1	3/2	3/1	3/1
7	1/1	5/3	2/2	3/3	1/1	1/1	4/2
8	1/1	1/1	1/1	1/1	2/1	3/2	3/2
Mean difference (SD)	0	−0,5 (0,9)	−0,25 (0,46)	−0,38 (0,74)	−0,5 (0,53)	−0,5 (0,76)	−1 (0,76)
* p*-value	/	0,17	0,17	0,2	0,03	0,1	0,0007

**Table 3:** POSAS observer parameters assessed in the eight treated patients at T0 and T2

No.	Vascularity	Pigmentation	Thickness	Relief	Pliability	Surface area	Overall opinion
1	4/2	2/2	4/2	3/2	2/2	2/2	3/2
2	2/1	2/1	4/2	2/2	4/2	3/2	4/3
3	3/1	1/1	4/2	2/1	3/2	2/1	4/2
4	2/1	2/1	2/2	2/2	2/1	2/2	2/1
5	1/1	2/1	3/2	2/2	3/2	1/1	2/1
6	2/1	3/3	4/2	1/1	4/2	1/1	4/3
7	2/2	2/2	3/2	3/2	2/2	2/2	3/2
8	3/2	3/3	4/4	2/2	2/2	4/3	4/3
Mean difference (SD)	−1 (0,76)	−0,38 (0,52)	−1,25 (0,89)	−0,38 (0,52)	−0,88 (0,83)	−0,38 (0,52)	−1.1 (0,35)
* p*-value	0,007	0,08	0,005	0,08	0,02	0,08	0,00004

**Table 4:** Antera evaluation: Scar depressions (mm 3) assessed in the eight treated patients at T0 and T2

No.	Depression at T0 (mm 3)	Depression at T2 (mm 3)	Difference between depressions (mm³)	Percentage reduction
1	6,72	4,58	−2,14	−31,8%
2	9,15	6,79	−2,36	−25,8%
3	4,11	2,69	−1,42	−34,6%
4	9	4,7	−4,3	−47,8%
5	8,23	7,3	−0,93	−13,5%
6	3,45	3,18	−0,27	−9%
7	4,18	3,5	−0,68	−16,3%
8	8,36	7,57	−0,79	−9,4%
Mean (SD)	6,65 (2,39)	5,04 (1,94)	−1,61 (1,3)	−23,5%
* p*-value			0,01	

Following treatment, all patients experienced erythema and edema, which both went away in the first seven days without any further issues. There were no additional potential side effects, including the development of blisters, viral or bacterial infections, post-inflammatory hyperpigmentation, or scar hypertrophy.

## Discussion

Surgical scars are a common outcome of many medical procedures and can have aesthetic, functional, and psychological implications for patients. The use of fractional CO₂ laser has established itself as one of the most promising options for scar treatment, thanks to its ability to improve skin quality and reduce the visible signs of scar lesions.

Surgical scars can be treated with various types of lasers, depending on the healing stage, scar type, and desired outcome.

 The Dye Laser (585–595 nm), which selectively targets hemoglobin, is especially effective in reducing redness in vascular or hyperemic scars during early stages. It is also used to treat or prevent hypertrophic scars and keloids, particularly in patients with a positive family history [23–25].

 Another widely used selective laser that emits in the near-infrared range is the Nd: YAG 1064 nm. In its Q-switched mode, it acts selectively on melanin and is effective in improving post-scar pigmentation, such as post-surgical hyperpigmentation [26].

Non-selective lasers, such as CO₂ and Erbium lasers, target water as their main chromophore. Since water is present in all cells, these lasers do not differentiate between tissue types and produce a more generalized effect on any material that absorbs their energy. Among non-selective lasers, we distinguish between ablative and non-ablative types.

 The CO₂ laser generates controlled ablation of the skin, resulting in the removal of damaged tissue and stimulation of new collagen production [27–29]. The CO₂ laser can be used in a traditional mode (single beam) or fractional mode (multiple microbeams). The traditional mode is useful for reshaping localized thickened scars, such as “trapdoor” scars. When used in pulsed mode, the laser delivers short, controlled bursts of energy, reducing thermal damage to surrounding tissue.

 However, traditional use causes a more significant disruption of the epidermis, leading to longer recovery times and a higher risk of complications compared to the fractional mode [30, 31].

 The 2940 nm Erbium: YAG laser is widely used worldwide for treating surgical scars [32]. Its wavelength matches the peak absorption of water, making it 12 times more absorbable than the CO₂ laser. As a result, it produces almost purely ablative effects with minimal thermal damage, reducing harm to surrounding tissue and allowing for faster wound healing and dermal recovery compared to the CO₂ laser [33]. Due to its high water absorption, the Erbium: YAG laser expends its energy quickly in water-rich tissue, limiting its action to the superficial skin layers. It cannot penetrate deeply into the dermis or effectively stimulate collagen, making it less suitable for atrophic scars but useful for very superficial scar resurfacing.

 Fractional CO₂ lasers can therefore be the ideal choice for individuals with more severe and deeper scars to achieve more significant results from the treatment [34].

 Non-ablative lasers, such as the 1540 nm Erbium: GLASS, can also be useful for certain types of scars. These lasers deliver energy that penetrates the epidermis without damaging it, reaching the dermis to stimulate collagen production through thermal effects without ablating tissue. They are particularly effective for atrophic scars, such as acne scars or atrophic surgical scars, with minimal side effects and short recovery times [35]. It is also possible to formulate a combined treatment protocol, alternating ablative and non-ablative lasers in order to stimulate the tissue with different wavelengths [36]. Overall, the choice between ablative and non-ablative laser systems should be guided by scar depth, morphology, and maturation stage, as ablative lasers provide more pronounced tissue remodeling at the cost of longer downtime, whereas non-ablative lasers offer a safer profile with milder but cumulative effects, supporting a tailored and stepwise therapeutic approach [37].

 Moreover, the concept of “fractional photothermolysis” has expanded the use of conventional lasers toward a new fractional mode, allowing for the gradual treatment of scars while significantly reducing unwanted side effects and broadening therapeutic options [38].

 Ablative fractional photothermolysis with a CO₂ laser induces the formation of microscopic zones of thermal damage within the epidermal and dermal layers; this mechanism leads to superficial epidermal ablation accompanied by thermal injury—via both ablation and coagulation—penetrating into the targeted skin layers, thereby effectively stimulating collagen production and dermal remodeling [39]. Only a biostimulatory effect is felt by the tissue surrounding each microcolumn, which is not impacted by the thermal damage brought on by coagulation and ablation [40].

The DEKA CO₂ laser features three delivery modes: Deep Pulse (DP), High Pulse (HP), and Smart Pulse (SP). DP uses longer, more intense pulses to penetrate deeper into the dermis, making it suitable for atrophic scars. HP delivers shorter pulses with greater ablative power, ideal for treating raised, superficial scars. SP represents a hybrid delivery mode between Deep Pulse and High Pulse, combining controlled ablative efficiency with a balanced thermal effect.

 This approach enhances biological effects on the tissue, stimulating collagen production and naturally improving skin texture, tone, and smoothness [41]. Therefore, when used in fractional mode, provides excellent results in the treatment of both atrophic and hypertrophic or raised scars relative to the skin surface.

Specifically, in the case of a “trap-door” scar, which is the focus of our analysis, a combined approach is required: on one hand, ablation is needed to remove the raised flap tissue caused by edema; on the other hand, deep thermal stimulation is necessary to promote collagen production along the suture line, which is often depressed due to scar contraction. This allows different clinical conditions (raised or atrophic scars) to be treated with a single device, simply by adjusting the pulse shape.

 Hypertrophic scars result from dysregulated wound healing, involving excessive collagen production driven by pro-inflammatory cytokines and growth factors like TGF-β, along with an imbalance between pro-inflammatory and pro-resolving mediators [42]. Following fractional CO₂ laser resurfacing, the initiation of collagen remodeling and scar repair involves multiple molecular mediators, including tumor necrosis factor-α (TNF-α), heat shock proteins, transforming growth factor-β (TGF-β), and matrix metalloproteinases (MMPs) [27, 43].

 Numerous studies support the efficacy and safety of fractional CO₂ laser in treating surgical scars. Notably, a meta-analysis by Shen reviewing 10 clinical trials demonstrated a significant reduction in Vancouver Scar Scale (VSS) scores, particularly in vascularity, stiffness, and thickness [44].

 Most studies focus on the efficacy of CO₂ laser in preventing pathological scars, with few addressing the treatment of “trap door” deformities [13]. Many rely on subjective clinical scores like the VSS, which do not provide quantitative measures of improvement. To overcome this limitation, our study used the Antera 3D device to objectively assess scar depression reduction. While some studies employ Antera 3D to evaluate CO₂ laser effectiveness on surgical scars, none have specifically focused on scars in the nasal subunit [45, 46]. This anatomical area, due to its characteristics, carries a higher risk of developing lymphatic stasis, flap edema, and consequently, a “trap-door” effect [13].

Our study assessed the efficacy of fractional CO₂ laser for treating post-surgical nasal scars, using the POSAS score for qualitative assessment and Antera 3D to quantify scar depression.

Eight patients with atrophic or “trap door” scars underwent two sessions spaced two months apart. Two months after the last treatment, POSAS scores showed statistically significant improvement reported by both patients and observers, with local side effects resolving within a week.

 Antera 3D analysis revealed an average 23.5% reduction in scar depression, slightly lower than the 38% reduction reported by Weiss et al. 2010 [47], likely due to differences in scar types, number of sessions, laser fluence, and longer follow-up in their study.

 It is interesting to note that in our study, the patients who showed the most marked improvements were those who had undergone surgery more recently. This finding suggests that in the first few months following surgery, the scar exhibits greater plasticity, responding more effectively to thermal and microablative stimuli. This observation aligns with studies showing that early treatments can prevent pathological scars and enhance aesthetic outcomes [48, 49].

 A recent meta-analysis of 14 trials involving 492 patients demonstrated significantly greater efficacy of fractional CO₂ laser treatment when performed within the first month after surgery [50]. A recent review also confirms that laser interventions are more effective when applied during the early stages of wound healing [51].

The main limitations of our study are the short follow-up period, which does not allow a robust assessment of long-term scar maturation and stability of outcomes, and the small sample size. In particular, the inclusion of only eight patients limits the statistical power of the paired t-test analysis, which should therefore be considered exploratory and not sufficient to support definitive conclusions. The absence of a control group represents an additional limitation.

In conclusion, fractional CO₂ laser remains an effective and safe treatment for improving surgical scars in the nasal region, showing significant results both in reducing scar depression and in the overall assessment of scar quality. However, further larger studies with longer follow-up, preferably designed as case-control trials, are needed to fully validate this conclusion and confirm the results obtained.

## Data Availability

No datasets were generated or analysed during the current study.
